# Preeclampsia and eclampsia-specific maternal mortality in Bangladesh: Levels, trends, timing, and care-seeking practices

**DOI:** 10.7189/jogh.13.07003

**Published:** 2023-07-14

**Authors:** Shusmita Khan, Abu Bakkar Siddique, Sabrina Jabeen, Aniqa Tasnim Hossain, M Moinuddin Haider, Fatema Tuz Zohora, Md Mahabubur Rahman, Shams El Arifeen, Ahmed Ehsanur Rahman, Kanta Jamil

**Affiliations:** 1 Data for Impact, University of North Carolina at Chapel Hill, North Carolina, USA; 2 International Centre for Diarrhoeal Disease Research, Dhaka, Bangladesh; 3 Independent consultant, Melbourne, Australia

## Abstract

**Background:**

Despite improvements in many health indicators, maternal mortality has plateaued in Bangladesh. Achieving the global target of reductions in maternal mortality and the associated Sustainable Development Goals will not be possible without actions to prevent deaths due to preeclampsia/eclampsia. Here we examined the levels, trends, specific causes, timing, place, and care-seeking behaviours of women who died due to these two causes.

**Methods:**

We used nationally representative Bangladesh Maternal Mortality and Health Care Surveys (BMMSs) conducted in 2001, 2010, and 2016 to examine levels and trends of deaths due to preeclampsia/eclampsia. We based the analysis of specific causes, timing, and place of preeclampsia/eclampsia deaths, and care seeking before the deaths on 41 such deaths captured in the 2016 survey. We also used BMMS 2016 survey verbal autopsy (VA) questionnaire to highlight stories that put faces to the numbers.

**Results:**

The preeclampsia/eclampsia-specific mortality ratio decreased from 77 per 100 000 live births in the 2001 BMMS to 40 per 100 000 live births in the 2010 BMMS, yet halted in the 2016 BMMS at 46 per 100 000 live births. Although preeclampsia/eclampsia accounted for around one-fifth of all maternal deaths in the 2010 BMMS, in the 2016 BMMS, the percentage contribution reached the 2001 BMMS level of 24%. An analysis of the VA questionnaire’s open section showed that almost all such death cases left their homes to seek care; however, most had to visit more than one facility before they died, indicating an unprepared health system.

**Conclusions:**

A cluster of preeclampsia/eclampsia-specific mortality observed during the first trimester, during delivery, and within 48 hours of birth indicates a need for preconception health check-ups and strengthened facility readiness. Awareness of maternal complications, proper care seeking, and healthy reproductive practices, like family planning to space and limit pregnancy through client-supportive counselling, may be beneficial. Improving regular and emergency maternal services readiness is also essential.

Preeclampsia and eclampsia are part of the hypertensive disorders spectrum that can occur during pregnancy, delivery, and postpartum periods [1]. For any previously known normotensive pregnant woman with 20 weeks gestation, the American College of Obstetricians and Gynecologists established criteria with nine symptoms for diagnosing preeclampsia, including high blood pressure (BP), proteinuria, oliguria, and cyanosis (**Box 1**). A woman with preeclampsia with seizures or convulsions is considered to have eclampsia [2]. Without adequate and timely treatment, preeclampsia/eclampsia can cause preterm birth due to convulsion, cerebrovascular accident, renal failure, haemolysis, elevated liver enzyme, low platelet count syndrome, disseminated intravascular coagulation, hepatic failure, coma, or even death of the mother and/or the foetus [3,4].

Box 1 **Diagnostic criteria of preeclampsia**

- Systolic BP ≥160 mmHg or diastolic BP ≥ 110 mmHg on two occasions six or more hours apart in a pregnant woman who is on bed rest
- Excretion of ≥5gm of protein in a 24-hour urine specimen or ≥3+gm on two random samples collected ≥4 hours apart – known as proteinuria
- Excretion of <500 mL of urine in 24 hours – known as oliguria
- Pulmonary oedema or cyanosis
- Impairment of liver function
- Visual or cerebral disturbances
- Pain in the epigastric area or right upper quadrant
- Decreased platelet count
- Intrauterine growth restriction

The United Nations (UN) Sustainable Development Goal 3.1 aims to reduce the global maternal mortality ratio (MMR) to less than 70 per 100 000 live births by 2030 [5]. Most developed countries have achieved this target. Although maternal mortality declined in recent decades in low- and middle-income countries (LMICs), the MMR remains high in many countries in sub-Saharan Africa and Southern Asia (eg, 480 per 100 000 live births in India, 897 per 100 000 live births in Myanmar in 2013) [6]. The high-MMR LMICs have been struggling to prevent two fatal yet preventable causes of death-haemorrhage and preeclampsia/eclampsia (also known as hypertensive disorder during pregnancy), while also bearing a high burden of indirect maternal deaths. The next level of decline warrants cause-specific programmatic actions [7-9].

Despite a steady decline in global maternal mortality, preeclampsia/eclampsia remains one of the most common reasons for maternal deaths, with 14% of all global maternal deaths caused by hypertensive disorders. This accounts for an estimated 343 000 annual deaths globally, with most occurring in developing regions (341 000 annual deaths), primarily in sub-Saharan Africa (209 000 annual deaths) and Southern Asia (80 000 annual deaths) compared with developed regions (1900 annual deaths) [10].

Based largely on clinical and hospital-based data, the incidence of eclampsia ranges between 2% and 10% in Southeast Asian countries like India, Pakistan, and Nepal. However, these hospital studies likely suffered from selection bias based on the severity of the condition, meaning that women with complications were more likely to visit facilities to seek health services. For example, in a hospital-based study in Bangladesh conducted between 1998 and 2000, among 32 999 obstetric patients, 2956 were eclamptic, yielding an incidence of 9% [11]. Despite being a major cause of maternal mortality, the prevalence of preeclampsia/eclampsia is not well documented, and the associated data are scarce in Bangladesh.

Since the early 1990s, emergency obstetric care and one-stop essential services package approaches have dominated maternal health in Bangladesh, with a focus on initiatives for both the demand (by creating awareness of facility-based delivery) and the supply side (by renovating and upgrading existing facilities and training facility staff) [12]. These initiatives resulted in a 40% decline in maternal mortality from 2001 to 2010 [13]. However, the overall maternal mortality rate has stalled since 2010 [14], and it seems like the “low-hanging fruits” of averting maternal mortality have already been achieved. Evidence suggests that, in countries like Bangladesh, delays in decision-making, transport, triage, and treatment occurring at the community, primary health centre, and hospital facility levels, respectively, contribute to maternal deaths due to time-sensitive cases like preeclampsia/eclampsia [15]. However, to the best of our knowledge, no study using national data comprehensively explored the care-seeking behaviour and the timing of maternal deaths due to preeclampsia/eclampsia to highlight the possible preventive and management measures.

Looking at the possible stagnation of the MMR in Bangladesh, the current task for Bangladesh’s maternal health sector is to renew national focus and revisit its program planning to prioritise interventions targeting the prevention and management of preeclampsia/eclampsia. This would require a critical understanding of the current situation and bottlenecks related to awareness, access, care-seeking practices, and quality of care for preeclampsia/eclampsia. We aimed to address the key evidence gaps and present the current level and trends in preeclampsia/eclampsia-specific maternal mortality in Bangladesh and also explore care-seeking practices before death, place of seeking service(s) before death (public and private sector facilities), and real-life stories to explain the causes behind the deaths.

## METHODS

We performed a secondary analysis of publicly available data from three rounds of the Bangladesh Maternal Mortality and Health Care Survey (BMMS), which used a nationally representative sample with a multi-stage cluster sampling procedure to measure the MMR with a three-year recall period [13]. The BMMS is conducted by the National Institute of Population Research and Training (NIPORT) of the Government of Bangladesh, with technical and financial assistance from the United States Agency for International Development (USAID) through the Demographic and Health Surveys (DHS) program (for the 2001 BMMS) and the Monitoring and Evaluation to Assess Use Results (MEASURE Evaluation) project (for the 2010 and 2016 BMMS). The first round of BMMS was conducted in 2001 [16] covering 99 202 households: the second round in 2010 [13] with 168 629 households, and the third round in 2016 [14] with 298 284 households (314 637 women ages 13-49 years and 81 284 live births in the three years preceding the survey).

Trained data collectors conducted verbal autopsy (VA) interviews using the standard World Health Organization (WHO) VA tool [17] adapted for Bangladesh. Trained physicians reviewed the VA forms and assigned a cause of death using the International Classification of Disease (ICD) codes [18]. We grouped similar ICD codes related to preeclampsia/eclampsia together and presented them as a broad category **(**Table S1 and S2 in the **Online Supplementary Document**). More details on the study design, sample size, sampling, and cause of death assignment process are available in the final reports of all three BMMS rounds [13,14,16].

### Analysis plan

We used Stata version 14 for the data analysis [19] and descriptive statistics to report preeclampsia/eclampsia-specific mortality fractions (presented as the percentage of total maternal deaths) and preeclampsia/eclampsia-specific mortality ratios (presented per 100 000 live births) with 95% confidence intervals (CIs) for the 2001, 2010, and 2016 BMMS, accounting for the complex sample design. We also presented the cause-specific mortality ratios stratified by several background, regional, and socioeconomic characteristics to explore potential determinants and changes across the three BMMS. However, the sample was not powered for this level of analysis; the findings stratified by several background, regional, and socioeconomic characteristics were merely indicative of what the situation in the field was likely to be.

We estimated the total number of preeclampsia/eclampsia-related maternal deaths per year using the total population reported in the 2011 national census report [20], the average annual growth rate reported by the World Bank to project the population in 2016 [21], crude birth rate reported in the 2016 BMMS to estimate the number of live births in 2016 [14], and the preeclampsia/eclampsia-related mortality ratio based on 2016 BMMS data [14]. We calculated uncertainty ranges (URs) using the CI obtained for the preeclampsia/eclampsia-specific mortality ratios.

We conducted the analyses on the timing and place of death, and care-seeking before death using 2016 BMMS data. We presented the timing of death by three broad categories: during pregnancy (first trimester, second trimester, third trimester), during delivery (after labour pains but before birth), and after delivery (day 0, day 1, day 2, days 3-6, days 7-42, and ≥43 days). We categorised place of death as home, health facility (public or private), and in transit (deaths that took place while traveling from home to a health facility, from one facility to another, or from a facility to home). We also presented the care-seeking patterns with descriptive statistics. Public facilities included district hospitals, maternal and child welfare centres, Upazila health complexes, and union health and family welfare centres. Private facilities included private hospitals and clinics.

We present the levels, causes, place, and care-seeking statistics in percentages. However, the sample size for preeclampsia/eclampsia was small (n = 41), so the findings were mostly indicative of the actual situation rather than statistically significant meaning that the small denominator-based percentages should be interpreted with care.

The novel contribution of this study, along with the quantitative analysis, is our investigation of the stories behind each death. Although the 2016 BMMS did not collect qualitative data using in-depth interviews or other methods for understanding the deep-rooted supply and demand-side barriers of maternal deaths, the open history section of the VA questionnaire narrated by the respondents (who were close to the deceased woman during her last days) provided an opportunity to identify barriers in maternal death prevention. We examined the VA open histories to identify incidences that described potential barriers in care seeking and care provided to the women during their last days. We then presented some stories with corresponding statistical figures. We randomly changed ages in single years, geographic identifiers, and details of children’s ages to avoid the risk of deductive disclosure from names and details in the stories. Moreover, only team members had prior approval to access the data to read the open sections and compile the stories.

### Ethical approval

We used publicly available data from NIPORT, Bangladesh, and the MEASURE Evaluation project for this analysis [22,23]. For the secondary analysis, we obtained ethical approval from the Research Review Committee (RRC) and Ethical Review Committee (ERC) of the International Centre for Diarrhoeal Disease Research, Bangladesh (icddr,b) because the secondary analysis involved the open section of the VA for proxy qualitative findings around the incidents that happened around the time of death.

## RESULTS

**Figure 1:**
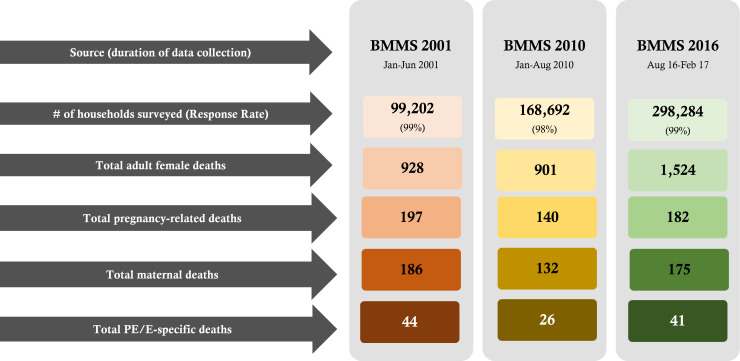
Number of preeclampsia/eclampsia-specific maternal deaths identified in 2001, 2010, and 2016 BMMS.

There were 53 805, 64 529, and 99 475 live births in 2001, 2010, and 2016, respectively, according to the BMMS conducted in those years (**Figure 1**). There were 493 maternal deaths among these live births, 111 of which were due to preeclampsia/eclampsia.

Table S3 in the **Online Supplementary Document** presents the background characteristics of the women who died due to preeclampsia/eclampsia-related complications during pregnancy, birth, or in the postpartum period in the 2001, 2010 and 2016 BMMSs. We included 41 deaths due to preeclampsia/eclampsia identified during the 2016 BMMS in the analysis and estimates. Almost three-fourths (76%) of the deaths due to preeclampsia/eclampsia in the 2016 BMMS took place in rural areas. More than 59% were in families with lower socioeconomic conditions.

### Did we make any progress across the survey years?

**Figure 2:**
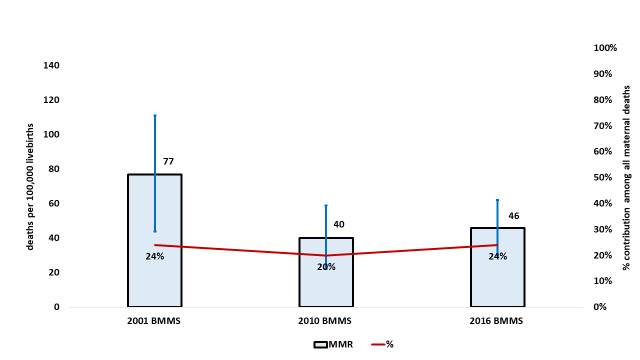
Levels and trends in preeclampsia/eclampsia-specific maternal deaths in Bangladesh, presented as the number of deaths per 100 000 live births and percentage contribution of all maternal deaths.

Preeclampsia/eclampsia-specific mortality ratio changed from 77 (UR = 44-111) per 100 000 live births in the 2001 BMMS to 40 (UR = 19-60) per 100 000 live births in the 2010 BMMS. However, it seems that the trend reversed in the 2016 BMMS to an MMR of 46 (UR = 29-65) per 100 000 live births. Although preeclampsia/eclampsia accounted for around one-fifth of all maternal deaths in the 2010 BMMS, the percentage contribution in the 2016 BMMS went up to the 2001 BMMS level of 24%. However, these changes were not statistically significant (**Figure 2**).

### Who were these women?

**Figure 3:**
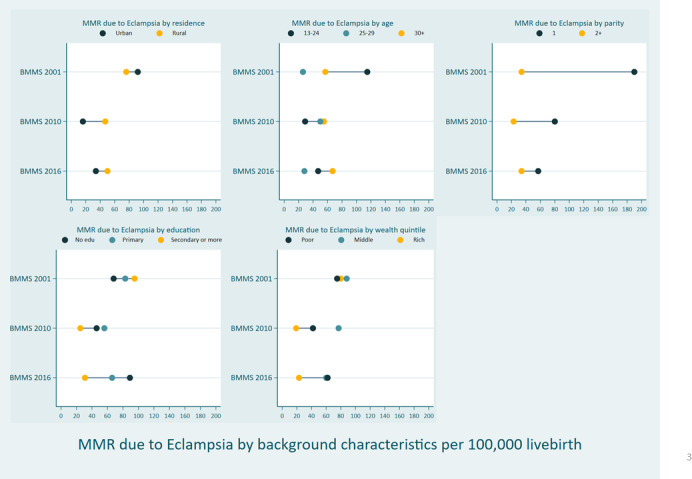
Preeclampsia/eclampsia-specific MMR by background characteristics and BMMS rounds, presented as the number of deaths per 100 000 live births.

In the 2001 BMMS, the preeclampsia/eclampsia-specific MMR was higher in urban areas; however, since that time, we observed a reverse trend in the 2010 and 2016 BMMS. Between the 2001 and 2010 BMMS, we observed a sharp decline in preeclampsia/eclampsia-related deaths in rural areas, but not for the post-2010 BMMS period. There has been no change in women aged ≥25 across surveys. However, among younger women (age <25 years), preeclampsia/eclampsia-related deaths declined between the 2001 and 2010 BMMS, but then they plateaued. Regarding parity, the preeclampsia/eclampsia-related deaths declined among primi women; however, they remained almost identical among multiparous women. Except for women with “secondary or higher” education, little difference was observed in terms of “educational attainment.” Last, across the surveys, MMR declined among the richer quintiles and remained similar for the bottom quintiles (**Figure 3**). Table S4 in the **Online Supplementary Document** provides additional details, with 95% CI. However, these changes were not statistically significant, just indicative of the trend.

### How many women died and where?

**Figure 4:**
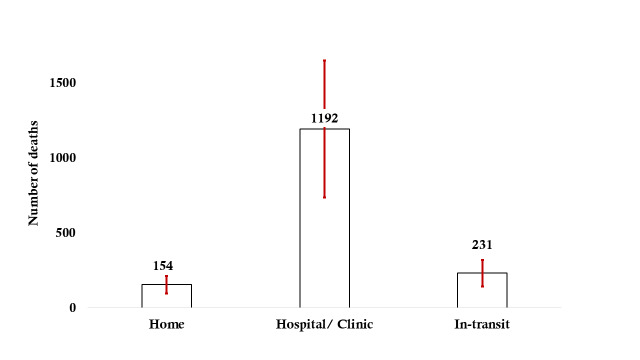
Estimated number of preeclampsia/eclampsia -specific maternal deaths per year in Bangladesh.

Each year, approximately 1577 (UR = 973-2181) women die due to preeclampsia/eclampsia-related complications during pregnancy, birth, and in the postpartum period, of whom 154 (UR = 95-213) died at home, 1192 (UR = 735-1649) at hospitals or health facilities, and 231 (UR = 143-319) in transit (**Figure 4**, **Box 2** and **Box 3**).

Box 2 **Who is to blame?**

*Sabina, a 24-year-old mother of two children, was pregnant with her third child and lived in an Upazila town with her husband and in-laws. From her first trimester, she had symptoms of preeclampsia. However, she did not go for any checkups or treatment because her previous two births were home-based with no negative incidence.*

*When Sabina was in her eighth month of pregnancy, one day she started having severe headaches, blurred vision, palpitations, respiratory distress, and a slight fever. This time her family got a little worried and took her to a qualified provider’s private chamber. The provider checked Sabina’s BP and then asked her to visit his private clinic the following morning. The next morning, Sabina’s family took her to the provider-suggested clinic and waited there until late at night for the provider to treat her. At around midnight, Sabina started to become very restless, and the provider was still not at the facility. Then Sabina’s family decided to transfer her to the district hospital.*

*At the district hospital, there was no one to manage her condition; the junior doctor referred Sabina to the nearest public medical college hospital. In the ambulance, Sabina started to convulse. The public medical college hospital was still quite far. The family then rushed the ambulance to the nearest private medical college. hospital. Sabina died in the emergency department of the private medical college hospital while convulsing.*

Box 3 **Everything that could go wrong, went wrong**

*Rina (29) a schoolteacher, pregnant with her second child lived in an urban area with her husband. During her first pregnancy, Rina had a history of high BP and was told by the doctor that she was hypertensive. From her second trimester, Rina started experiencing blurred vision. Rather than using her regular medication, Rina measured her BP at a nearby pharmacy and took medicines on her own.*

*Just two weeks before her expected due date on a Thursday, Rina woke up with severe headache and slight breathing difficulty. Her husband was at work, so Rina took a three-wheeler and went to the nearby clinic. The duty doctor found her BP to be very high and advised for admission. On Rina’s husband’s arrival, they decided to go to her usual doctor. By the time they reached the other clinic, the usual doctor had left the clinic. Because Rina was feeling better, they decided to stay for the night under observation and go for an elective Caesarean section (C-section) on Saturday.*

*At around midnight, Rina became restless and there was no doctor to manage the situation. Rina’s husband arranged an ambulance and rushed her to another facility. The ambulance reached another clinic, but they refused to admit her because the clinic did not have anyone to manage her situation. At this point Rina started to convulse, so the ambulance rushed again, trying to reach the most expensive and highly equipped private hospital in the town. Rina died on the way.*

### Mapping the deaths

**Figure 5:**
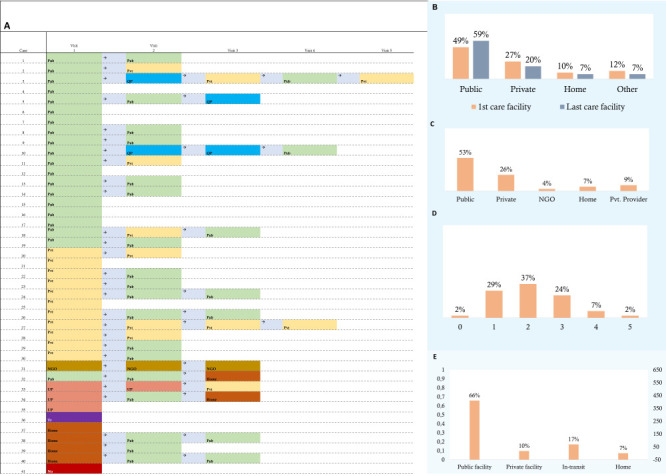
Place of care seeking and death due to preeclampsia/eclampsia, 2016 BMMS. **Panel A.** Care-seeking trajectory and places. **Panel B.** Care-seeking summary of Panel A. **Panel C. **Percentage distribution of place of care-seeking visits (total visits, n = 85). 41 women who died due to preeclampsia/eclampsia sought care a total of 85 times. **Panel D. **Percentage distribution of frequency of care-seeking visits (total visits, n = 41). **Panel E**. Percentage distribution of place of death due to preeclampsia/eclampsia, 2016 BMMS (n=41). Pub – public hospital, pvt – private facility, home – at home by (un)qualified provider, QP – qualified private practitioner (PP) at his/her place, UP – unqualified PP at his /her place, NGO – non-governmental organisation’s facilities, Tr – in transit, NO – no service sought.

We mapped the journey that each woman made before dying due to preeclampsia/eclampsia in the 2016 BMMS (**Figure 5**). Most women who died of preeclampsia/eclampsia in the 2016 BMMS (n/N = 35/41) sought care from a health facility at least once. With one case not seeking any care, the 40 women who died due to preeclampsia/eclampsia in the 2016 BMMS made a total of 85 visits to different facilities, with an average of 2.1 visits to different facilities. More than one-third (37%) of the women visited two facilities, one-fourth (24%) visited three facilities, and 9% visited four or more before dying due to preeclampsia/eclampsia (**Figure 5**, panel D). Although 27% of the women went to private facilities (**Figure 5**, panel B) as their first point of contact, only 10% died at private facilities (**Figure 5**, panel E) (**Box 4**).

Box 4 **The passage of death**

*Around two weeks before Khadija’s death (at age 30), she experienced severe headaches followed by profuse vomiting episodes of more than five times. Seeing her agony, the family took unconscious Khadija to a private clinic where she started to bleed. The clinic did not have proper treatment facilities or a skilled provider to handle her case; therefore, she was referred to the public medical college hospital.*

*Two days after reaching the public medical college hospital, unconscious Khadija gave birth to a stillborn baby through C-section. After the birth, Khadija regained consciousness, but bleeding continued. After three more days of bleeding and weakness, Khadija became unconscious and was referred to the divisional public medical college hospital for intensive care unit (ICU) support.*

*After reaching the divisional public medical college hospital, the family could not find a place in the ICU and took unconscious Khadija to a private hospital for ICU support. At that facility, she received dialysis support and two bags of blood as she continued to bleed.*

*After seven days in the ICU, the family decided to shift Khadija to a public facility because it was hard for the family to bear the cost of treatment. At the public facility, Khadija died waiting for a bed in the ICU.*

### When were these women dying?

**Figure 6:**
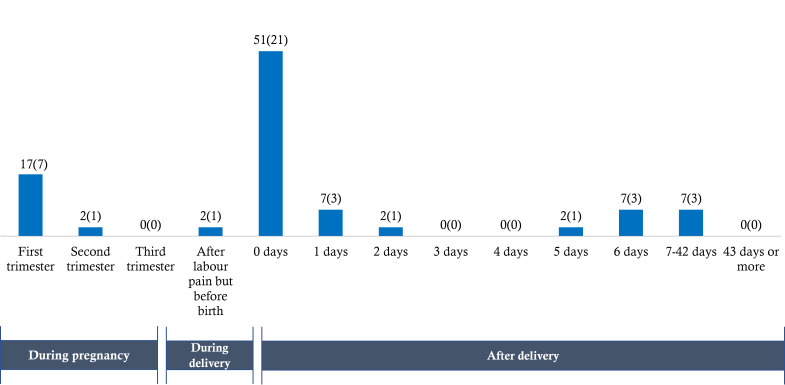
Timing of preeclampsia/eclampsia-specific maternal deaths (n = 41), presented as percentage distribution.

**Figure 6** shows the timing of preeclampsia/eclampsia-specific maternal deaths in the 2016 BMMS. Half (n = 21, 51%) of the deaths due to preeclampsia/eclampsia-specific maternal causes happened during and within 48 hours of delivery. Eight (19%) of the deaths took place before delivery, with the majority happening during the first trimester (**Box 5**) (**Box 6**)

Box 5 **Died too young**

*Peyara was just 17 years old when she got pregnant with her first child. During the first trimester, she always complained about headache and used to drink unusual amounts of water.*

*Three days before her death, when she was just entering the fourth month of her pregnancy, she complained about excessive headache and blurred vision and decided to bathe. When she was entering her room after bathing at the nearby pond, she felt nauseated and lost consciousness.*

*When her husband tried to pick her up, he noticed that Peyara was bleeding and had fecal matter on her dress. The family rushed her to a nearby hospital thinking she might have a miscarriage.*

*At the hospital, the doctor controlled her bleeding, but she started having convulsions and she was referred to the public medical college hospital for further treatment. Peyara died in the eclampsia-ward of the public medical college hospital.*

Box 6 **Mother of a daughter**

*All Bilkis (32) ever wanted was a son. She gave birth to three daughters, and this was her fifth pregnancy. Her fourth pregnancy was terminated because ultrasonography revealed that she was having a daughter.*

*When she was nearing her term, she was sent to her parent’s home for delivery services because she needed a C-section and her husband refused to pay for the cost. Her parents were not well off, therefore Bilkis decided to try for a home delivery.*

*After 21 hours of labour, the dai (traditional/untrained birth attendant) asked to take her to a hospital because Bilkis was shivering (convulsing). After reaching the government facility, the family found that the facility did not have a provider or medicine to handle her convulsions or the birth. She was referred to another hospital.*

*The family put her on a paddle-van and took her to the medical college hospital. After reaching there, she was rushed to the operating room for a C-section.*

*Bilkis died within two hours of giving birth to a baby boy.*

## DISCUSSION

We documented the details of preeclampsia/eclampsia-related maternal deaths in Bangladesh. Overlapping URs of preeclampsia/eclampsia-specific MMR between the 2010 and 2016 BMMS showed a trend of stagnation. As in the 2010 BMMS, preeclampsia/eclampsia-specific deaths remained slightly concentrated among underprivileged (from rural and poor households) sociodemographic groups in the 2016 BMMS. The women's stories reflect demand-side urgent care seeking when experiencing life-threatening events and failure to receive life-saving emergency services on reaching medical facilities. Deaths due to preeclampsia/eclampsia mostly occurred around the day of delivery and in the first trimester.

Bangladesh made remarkable advances in reducing maternal mortality between 2001 and 2010. A sharp decline in eclampsia and haemorrhage-specific deaths helped reduce the MMR during this period [10], possibly due to the increased coverage of antenatal care (ANC) uptake and facility-based births [24-26]. Despite the continuous progress in ANC and facility-based delivery coverage, maternal mortality remained stagnant in 2016 at the 2010 level. This implies that the existing maternal healthcare service system in Bangladesh has successfully prevented the low-risk maternal deaths but failed to manage critical and emergency obstetric situations. The unchanged share of preeclampsia/eclampsia in maternal mortality during the past decade shows that cause-specific interventions to reduce preeclampsia/eclampsia-related deaths have not worked as expected [10,13,27]. A further decrease in deaths due to preeclampsia/eclampsia will require segmented and targeted interventions, like prompt identification and management with anticonvulsants. This can only happen if facilities possess the right equipment, medicines, an onsite (not on call) skilled provider, and effective referral mechanisms (data not published). This would include revision of existing policies, strategies, and service protocols to prioritise the handling of preeclampsia/eclampsia-related cases with available resources [28,29].

Our findings reveal that, in some cases at the client level, decisions were taken with a slight delay, which often led to delay in appropriate, timely treatment. We observed that decisions were delayed at different stages of maternity – during pregnancy, labour, and postpartum. With targeted, timely efforts, most of these preeclampsia/eclampsia deaths could be prevented.

### Preconception and early stage of conception

The 2017-2018 Bangladesh Demographic and Health Survey revealed that 51% of women ages 18 years and above were unaware of their raised BP status [30]. Undiagnosed raised BP may be aggravated immediately after conception and delays in seeking first ANC may intensify the severity. Despite many challenges, the Government of Bangladesh has successfully generated demand for ANC uptake across sociodemographic groups. However, the timing of seeking the first ANC is a crucial topic, because ANC in the first trimester is low, while the first trimester is one of the sensitive times for preeclampsia/eclampsia deaths. About half the women who received ANC sought their first ANC visit after 4.7 months of pregnancy [30]. These two issues show such high levels of undiagnosed raised BP among adult women along with delayed first ANC can lead to an undiagnosed preeclampsia/eclampsia situation during pregnancy. Thus, health check-ups before conception and ANC in early stages of pregnancy can be beneficial in preventing deaths due to preeclampsia/eclampsia [31,32].

### The ‘’3E”

The “3E” – early identification, early diagnosis, and early treatment – are key to averting the severity of any health condition, including preeclampsia/eclampsia. Women who developed eclampsia experience a wide range of symptoms, starting with oedema, headache, blurred vision, and high BP. Despite the life-threatening chances of convulsion, two-thirds of women who had a live birth in the three years preceding the 2016 BMMS were unaware of its danger signs [11]. More than two-fifths of women with preeclampsia symptoms and 15% with convulsion symptoms did not seek treatment because most of them thought that the symptoms were not serious [11]. Although four of five women had at least one ANC visit from a medically trained provider, only about 40% of them were informed about the signs of pregnancy complications, signalling a high opportunity loss [33]. More than one-fourth of the women who sought ANC did not undergo a urine test, which is a medical diagnosis tool for eclampsia. Ignorance of these symptoms may lead to severe conditions that are primarily manageable if diagnosed in the early phase. Our results showed that 93% of eclampsia deaths took place at any health facility or in transit, which signifies care-seeking practices during terminal illness. This implies that if they could understand the severity, they were likely to seek institutional care. Therefore, counselling about eclampsia symptoms and its life-threatening risk during ANC could be the first step in preventing eclampsia deaths.

### During and after delivery

We found that 60% of eclampsia-specific deaths happened during and within 48 hours of birth. However, the 2016 BMMS reported a large demand-side gap in pre-arrangement of funds and transportation for emergencies. The findings showed that seven of 10 deceased women sought care from at least two facilities, highlighting a lack of community awareness about the health facilities where they can get services during emergency eclampsia conditions. ANC providers should inform women and their caregivers (could be the spouse or anyone from the family) where to go for treatment of eclampsia during an emergency. Proper counselling on birth preparedness and danger signs during pregnancy can be beneficial to overcome this demand-side barrier.

### Costs associated with preeclampsia or convulsion treatment

The VA open section of the 2016 BMMS showed that almost all preeclampsia/eclampsia-specific deaths that took place during and within 48 hours of delivery had convulsions before their deaths. Although our sample size was rather low (n = 41) because it came from a nationally representative survey, we can assume that the pattern found in our study would be somewhat closer to reality. The aetiology of convulsion is less explained, but evidence shows that convulsion causes cerebral haemorrhage, and 60% of the subjects expire within 48 hours of the convulsion [34]. The treatment costs of preeclampsia and convulsion were a leading reason for not seeking care [11]. We found a three times eclampsia-specific MMR among women from the third and lower wealth quintiles compared with women from the top two quintiles. Alleviating this socioeconomic inequity without reducing out-of-pocket expenditures would be cumbersome.

### Facility readiness is the key

The estimated number of deaths due to preeclampsia/eclampsia in 2021 shows that most of these deaths took place at facilities. We also found that even a small proportion of women who gave birth at home sought care from a facility, but unfortunately died. This matches the evidence from other countries with a lack of trained health providers, supply of medicines, and logistics at facilities like in Bangladesh [35-37]. Another important finding is the shuttling of critical cases. Our results showed that critical time was spent switching between facilities before dying. This raises questions about the readiness of facilities to manage preeclampsia/eclampsia conditions. According to the 2017 Bangladesh Health Facility Survey (BHFS), two-thirds of public facilities offering normal delivery services had no anticonvulsants, and one-third of private facilities did not have anticonvulsants, reflecting poor readiness to tackle emergency eclampsia cases [38]. The 2017 BHFS also found that only 14% of the district-level government facilities had injectable magnesium sulphate [38]. Moreover, the availability of magnesium sulphate has decreased markedly since 2014 [38]. This lack of readiness explains why women had to switch from one facility to another – revealing how unprepared the facilities were to provide appropriate care to manage preeclampsia/eclampsia, which is evident in the individual care-seeking flowchart presented here.

### Faulty referral system causes death

Possible reasons for multiple switching of facilities are not limited to inadequate readiness, but also include a fragile referral system. If a woman with eclampsia symptoms reaches a facility that is incapable of providing treatment, they are expected to refer her to another facility that can manage her condition and inform the referral facility about her current situation, which may ensure immediate treatment. In this manner, a properly functioning referral system can make the shuttling to another facility endurable. However, we found that one-third of the women who died due to preeclampsia/eclampsia switched facilities multiple times. Well-prepared health facilities that can manage emergency eclampsia conditions are not yet equitably distributed and it seems that ensuring an equitable distribution across all sociodemographic groups is still a distant target. By acknowledging this limitation, establishing an effective referral system could be a call to action to prevent preeclampsia/eclampsia-specific deaths with available resources.

### Strengths and limitations

The findings and the stories behind the deaths due to preeclampsia/eclampsia presented here were analysed using the most recently published data of the nationally representative 2016 BMMS [14]. This survey appropriately represents the sociodemographic characteristics of inhabitants from Bangladesh’ urban and rural areas by including samples from all of its administrative divisions. Therefore, the results can be considered generalisable for maternal mortality due to preeclampsia/eclampsia in Bangladesh. The VA tool was developed and finalised by adapting the WHO standard VA tool to the country context through a vigorous exercise by maternal and public health experts. The review physicians received intensive training on assigning cause of death and using the online ICD 10 classification of diseases. The study authors were involved in tool development, finalisation, training, and monitoring of the data collection team, and assigning the cause of death, adding quality and validity of the data we used in our analysis.

However, the secondary data used here may have possible sources of bias. Obtaining a primary source for such data are very resource-intensive. Although the survey samples were nationally representatives (2001, 2010, and 2016 BMMS), the preeclampsia/eclampsia case samples were small so there was low statistical power to draw conclusions. Disaggregated estimates by different background characteristics were not sufficient for gaining an acceptable level of precision. The VA method demands the collection of information with a lot of clinical symptomatic details from a close family member of the deceased mother, resulting in possible recall error and recall bias. Last, while we used data from the most recently published national maternal mortality surveys – BMMS 2001, 2010 and 2016 – the analysis of the open data section of the VA was only done for the BMMS 2016. We wanted to investigate the open sections for the VA for BMMS 2010 and 2001 too, but the hard copies of those responses were not available for analysis.

## CONCLUSIONS

Despite some improvements from the 2000s in reducing the MMR, Bangladesh has now reached a plateau. Preeclampsia/eclampsia has been a top cause of deaths since the 2000s and has always been flagged as a “low hanging fruit” in reducing MMR. However, we found that, despite improvements in reducing preeclampsia/eclampsia-related deaths from 2001 to 2010, the MMR stalled in 2016. Women went to facilities to seek care but still died, often after shuttling from one facility to another. Clear and consistent guidelines with comprehensive steps for implementation, and role clarification for health facilities and healthcare providers could ensure positive outcomes during the management of preeclampsia/eclampsia. In conjunction with guidelines, multidisciplinary and multisectoral approaches from the antenatal to the postnatal period could rapidly reduce these preventable deaths.

## Additional material


Online Supplementary Document
